# Correction to: CReSIL: accurate identification of extrachromosomal circular DNA from long-read sequences

**DOI:** 10.1093/bib/bbad402

**Published:** 2023-10-20

This is a correction to: Visanu Wanchai, Piroon Jenjaroenpun, Thongpan Leangapichart, Gerard Arrey, Charles M Burnham, Maria C Tümmler, Jesus Delgado-Calle, Birgitte Regenberg, Intawat Nookaew, CReSIL: accurate identification of extrachromosomal circular DNA from long-read sequences, *Briefings in Bioinformatics*, Volume 23, Issue 6, November 2022, bbac422, https://doi.org/10.1093/bib/bbac422

In the originally published version of this manuscript, there was an error in Panel E of Figure 3 due to a mistake in the script to plot the upset plots. This has been corrected in the original article.

In addition the following sentence was also incorrectly given as:

“However, we found over 90 000 recurrent eccDNAs between replicates of the ARP1 cell line (∼50%), indicating a cell-specific population of eccDNA that possibly benefits the ARP1 cell line (Figure 3E left panel)”

This has now been corrected to:

“However, we found over 28 000 specifically recurrent eccDNAs between replicates of the ARP1 cell line (∼15%), indicating a cell-specific population of eccDNA that possibly benefits the ARP1 cell line (Figure 3E left panel).”

These errors have been corrected in the original article.

